# Biomechanics and energetics of walking in powered ankle exoskeletons using myoelectric control versus mechanically intrinsic control

**DOI:** 10.1186/s12984-018-0379-6

**Published:** 2018-05-25

**Authors:** Jeffrey R. Koller, C. David Remy, Daniel P. Ferris

**Affiliations:** 1 Department of Mechanical Engineering, University of Michigan, 2350 Hayward, Ann Arbor, MI, 48109 USA; 2 J. Crayton Pruitt Family Department of Biomedical Engineering, University of Florida, 1275 Center Drive, Gainesville, FL, 32611 USA; 3 Department of Mechanical Engineering, University of Florida, 1275 Center Drive, Gainesville, FL, 32611 USA

**Keywords:** Exoskeleton Control, Gait, Kinematics, Power, Electromyography

## Abstract

**Background:**

Controllers for assistive robotic devices can be divided into two main categories: controllers using neural signals and controllers using mechanically intrinsic signals. Both approaches are prevalent in research devices, but a direct comparison between the two could provide insight into their relative advantages and disadvantages. We studied subjects walking with robotic ankle exoskeletons using two different control modes: dynamic gain proportional myoelectric control based on soleus muscle activity (neural signal), and timing-based mechanically intrinsic control based on gait events (mechanically intrinsic signal). We hypothesized that subjects would have different measures of metabolic work rate between the two controllers as we predicted subjects would use each controller in a unique manner due to one being dependent on muscle recruitment and the other not.

**Methods:**

The two controllers had the same average actuation signal as we used the control signals from walking with the myoelectric controller to shape the mechanically intrinsic control signal. The difference being the myoelectric controller allowed step-to-step variation in the actuation signals controlled by the user’s soleus muscle recruitment while the timing-based controller had the same actuation signal with each step regardless of muscle recruitment.

**Results:**

We observed no statistically significant difference in metabolic work rate between the two controllers. Subjects walked with 11% less soleus activity during mid and late stance and significantly less peak soleus recruitment when using the timing-based controller than when using the myoelectric controller. While walking with the myoelectric controller, subjects walked with significantly higher average positive and negative total ankle power compared to walking with the timing-based controller.

**Conclusions:**

We interpret the reduced ankle power and muscle activity with the timing-based controller relative to the myoelectric controller to result from greater slacking effects. Subjects were able to be less engaged on a muscle level when using a controller driven by mechanically intrinsic signals than when using a controller driven by neural signals, but this had no affect on their metabolic work rate. These results suggest that the type of controller (neural vs. mechanical) is likely to affect how individuals use robotic exoskeletons for therapeutic rehabilitation or human performance augmentation.

**Electronic supplementary material:**

The online version of this article (10.1186/s12984-018-0379-6) contains supplementary material, which is available to authorized users.

## Background

When it comes to designing the control of lower extremity assistive robotic devices, such as exoskeletons or prostheses, there are a wide variety of control strategies to choose from. Ideally, with the correct control architecture and proper tuning, these devices can work in parallel with the user to aid in their locomotion [1–5]. There have been many different control strategies explored in research, but there is a lack of knowledge in knowing what type of control to use for certain applications and why.

Lower extremity robotic devices have traditionally been separated into two main approaches for device control. The device assistance can either be driven by *neural signals* or *mechanically intrinsic signals*. Control driven by neural signals relies on the already existing control architecture of the human body. By tapping into physiological electrical signals, such as brain activity or muscle activity, these controllers can decode human intention and actuate the device accordingly [6–8]. Control driven by mechanically intrinsic signals relies on measures that are intrinsic to the device itself, such as detected gait events, joint angles, or forces [9–12]. In doing so, these devices are trying to infer human intention from secondary information to drive actuation. For example, a joint angle may be used as a phasing variable for the onset of a predefined actuation signal [13].

Each of these control approaches has its own advantages and disadvantages. For example, control driven by neural signals is often argued to have better human-device synchronization over control driven by mechanically intrinsic signals [14]. Neural signals can be measured before force generation at the muscle has actually occurred due to the electromechanical delay of the body [15]. Therefore, there is a buffer of time between sensing of a neural signal and delivering actuation that is synchronous with the user’s movement. In contrast, mechanically intrinsic signals can only be sensed after movement has already occurred. This creates an inherent lag behind the user when using control driven by mechanically intrinsic signals, yet if designed properly this lag may be indistinguishable by the user [16, 17]. Another advantage for control driven by neural signals is that it can allow for direct control by the user. With proportionality in the control scheme, users can directly control the timing and amplitude of actuation at any time instance using the same neurological control they would adjust their own muscle contraction timing and amplitude. This proportionality can lead to a more natural means of control and adaptation compared to a controller driven by mechanically intrinsic signals [18]. One big advantage of using mechanically intrinsic signals to drive control is the reduced complexity over neural signals. Sensors used to measure mechanically intrinsic signals can be self-contained in the device and produce consistent and repeatable measurements. With neural signals, the electrodes used for electroencephalography (EEG) or electromyography (EMG) can have large variability depending upon placement and skin conditions. With relatively high noise content, neural signals require extensive decoding or filtering before they can be used in real time.

**Fig. 1:**
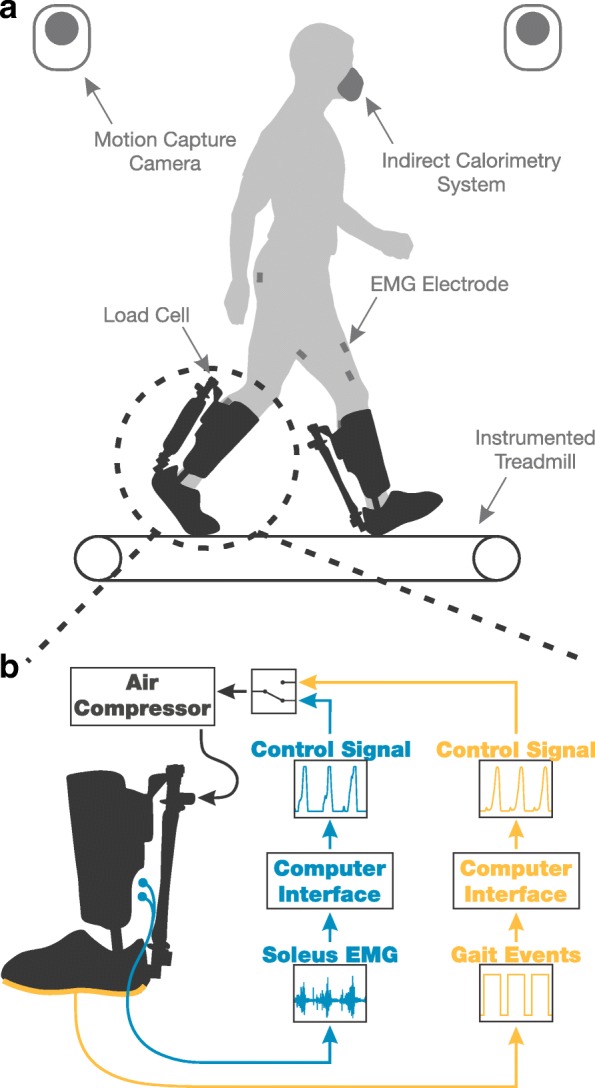
Experimental Setup and Control Schemes. **a** All eight subjects walked at 1.2 ms^-1^ with pneumatically powered bilateral ankle exoskeletons. During testing we measured subjects’ joint kinematics and dynamics (motion capture and instrumented treadmill), muscle activity (EMG electrodes), exoskeleton kinetics (load cells), and energy consumption (indirect calorimetry). **b** Subjects completed separate 10 minute walking trials with two different control schemes. The dynamic gain proportional myoelectric controller (blue) created a actuation signal proportional to subject’s soleus muscle recruitment. The timing-based mechanically intrinsic controller (yellow) sent through the same predefined actuation signal triggered by each heel strike. The two controllers were designed to have the same average actuation signal

Despite the prevalence of these two types of controller designs, to date, there does not exist any systematic and fair comparison of how they differently affect the biomechanics and energetics of individual users. In the work presented here, we designed an experiment to make a close comparison between a controller driven by neural signals (a proportional myoelectric controller based on soleus muscle recruitment) and a controller driven by mechanically intrinsic signals (a timing-based mechanically intrinsic controller based on gait events). We tested both controllers with healthy subjects wearing bilateral ankle exoskeletons and aimed to better understand users’ biomechanical and energetic responses to each during steady-state treadmill walking (Fig. 1). We designed these two controllers to have the *same average* actuation signal such that the main difference between them was the way in which the actuation was driven. To ensure the same average actuation signal, we created the actuation profile for the timing-based controller directly from the average of control signals seen during use with the proportional myoelectric controller. In previous work, we have mathematically derived the inherent relationship between muscle activation and device output when using a proportional myoelectric controller [19]. The timing-based controller we are showing here does not have such dependency so the actuation signal was consistently the same regardless of the user’s soleus muscle recruitment. Our primary hypothesis was that the human nervous system would identify this key difference between controllers and therefore use each in a distinct way, thus resulting in a difference in metabolic work rate between the two controllers. We have investigated where some of these differences in use may be by analyzing subject’s muscle recruitment, joint kinematics, and joint dynamics.

## Methods

### Subjects

In this study we tested eight healthy subjects with no prior experience walking in powered exoskeletons (male, 21 ± 1 years, 74.0 ± 2.7 kg, 180.0 ± 2.8 cm; mean ± standard error of the mean). We pre-screened all participants for exoskeleton hardware fit prior to testing. All subjects gave informed written consent to participate in the study in accordance to the University of Michigan Medical School’s Institutional Review Board.

### Exoskeleton hardware

The bilateral ankle exoskeletons that we used in this study were similar in design to our previous work [8, 20–22]; however, we designed these exoskeletons to be more adjustable and versatile to fit a number of subject sizes. These were the same exoskeletons as presented in [19].

The exoskeletons consisted of an adjustable shank component attached to a shoe component by a single degree of freedom rotational joint. The rotational joint constrained the exoskeleton’s motion to plantar flexion and dorsiflexion. The shank component was made from stainless steel rods and plastic cuffs. We used ratchet straps on the cuffs to fit the shank to different subject sizes. The shoe component was a standard orthotic shoe that we outfitted with metal attachments for actuation and joint coupling. The exoskeleton could accommodate subjects that wore between a 9 and 11 U.S. men’s shoe size.

We actuated the exoskeletons using custom built artificial pneumatic muscles attached posteriorly. These actuation units only provided plantar flexion assistance to the user [20]. We attached a load cell in series (Omega Engineering, Stamford, Connecticut) with the actuator to measure actuation kinetics. The shoe, shank, actuator, and load cell combined to a total mass of 2.08 kg (approximately 0.81 kg at the foot and 1.27 kg at the shank).

### Exoskeleton control

In this study we used two different controllers, a dynamic gain proportional myoelectric controller and a timing-based mechanically intrinsic controller, on the same exoskeleton hardware. We built both of these controllers in Simulink (The MathWorks, Inc., Natick, MA) and compiled them to run on a real-time control board (dSPACE, Inc., Northville, MI).

#### Dynamic gain proportional myoelectric control

The proportional myoelectric controller was driven by user’s soleus EMG activity. We measured subjects’ soleus activity in real time using EMG surface electrodes (sample rate: 1000 Hz; Biometrics, Ladysmith, VA). The designed controller then processed the recorded signal into its linear envelope by high-pass filtering (2nd order Butterworth, cutoff frequency 80 Hz), full-wave rectifying, and then low-pass filtering (2nd order Butterworth, cutoff frequency 4 Hz) the raw signal.

The controller multiplied the calculated linear envelope by a gain to linearly map the small voltage of the processed EMG signal into a larger control voltage that was sent to the pneumatic pressure control valves (MAC Valves, Wixom, MI). We applied a threshold to this control signal such that the commanded pneumatic pressure needed to be greater than 20 pounds per square inch (p.s.i) in order to actuate as the pneumatic muscles were pretensioned with this pressure to allow for a faster response time. The maximum output pressure of our pressure source was 90 p.s.i. and control signals were saturated beyond this point. The controller was designed to continuously tune the linear mapping gain on a subject-specific basis using a dynamically adaptive algorithm as described in [19]. This algorithm tuned the gain such that the average peak EMG signal over the previous 50 strides mapped to a desired maximum control signal voltage. We chose a desired maximum control voltage that resulted in the peak of the average control signals to be the maximum output pressure of the valves (90 p.s.i.). This created a controller that, on average, supplied maximal peak actuation to the user at the same moment when they reached their maximal peak soleus activity for that given stride. In this control scheme, the user could adapt their own muscle activity to whatever level they felt comfortable with, while still receiving maximal peak power output from the device.

#### Timing-based mechanically intrinsic control

**Fig. 2:**
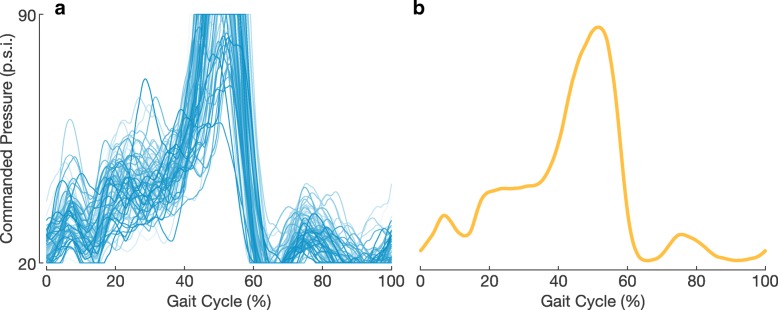
Creating the Timing-Based Control Signal for a Representative Subject. **a** The actuation signals from 80 of the final 100 strides of a subject’s walking bout with the dynamic gain proportional myoelectric controller were considered in creating the actuation signal for the timing-based controller. Those 80 strides are shown here for a single representative subject and from a single leg. The darker the color of the actuation signal, the later in the walking bout it occurred. **b** The actuation signal for the timing-based mechanically intrinsic controller was generated from the average of the 80 strides considered from the walking bout with the myoelectric controller

The timing-based mechanically intrinsic controller was driven by detected heel strikes as sensed by an instrumented treadmill (Bertec Corporation, Columbus, OH). We designed this controller to have the same average actuation signal as that of the proportional myoelectric controller (Fig. 2). To generate the actuation profile for this timing-based controller, we first normalized the actuation signals from the final 100 strides of a subject’s walking bout using the proportional myoelectric controller by their percent gait cycle (heel strike to heel strike). We then averaged these 100 normalized actuation signals. We calculated the root mean squared error (RMSE) for each of the 100 individual stride’s actuation signal compared to this average and then discarded the 20 strides with the largest RMSE values to safely remove any outliers. We then averaged the remaining 80 strides’ actuation signals to generate the actuation profile for the timing-based mechanically intrinsic controller. This whole process was performed separately for each individual subject and leg.

During walking, the timing-based controller would assist with plantar flexion upon each detected heel strike. This process was equivalent to pressing a “play” button on the predefined actuation signal with each heel strike. If a stride was shorter than the averaged control signal, the signal would start over immediately. If a stride was longer than the averaged control signal, the actuators remained at a pressure that resulted in zero force generation until the next detected heel strike occurred.

### Testing protocol

We trained all participating subjects in this study to walk with the powered ankle exoskeletons prior to the data collection presented here. All subjects had no experience with walking in a powered assistive device prior to this training. In recruiting a naive subject pool, we have ensured that all subjects were given the same amount of time to adapt and learn to walk in the exoskeletons. The training consisted of three separate days of walking with the exoskeletons using the dynamic gain proportional myoelectric controller. During these training sessions, subjects walked continuously on a treadmill at a fixed speed in the exoskeletons for 50 minutes, the middle 30 of which were powered. A more detailed description of these sessions and subjects’ adaptations is described in [19].

After completing the three training sessions, we tested subjects on a separate fourth day to collect the data presented here. During this final testing session, subjects participated in four walking bouts that were each 10 minutes long. Subjects were given a seated rest period of 5-10 minutes between bouts. First, subjects walked in the exoskeletons without any actuation. We will refer to this bout as the *unpowered* condition. Subjects then walked using the dynamic gain proportional myoelectric controller in order to re-familiarize themselves with the devices and to generate the data necessary to build the control signals for the timing-based mechanically intrinsic controller. This walking bout served purely as a warm up for subjects and a calibration for the timing-based controller. As such, no results from this bout are presented here. After the warm up session, subjects walked using the timing-based mechanically intrinsic controller and then walked using the dynamic gain proportional myoelectric controller. We will refer to these bouts as the *timing-based* and the *proportional myoelectric* controller conditions, respectively. All walking bouts took place at 1.2 m/s on an instrumented treadmill. We considered the final three minutes of each walking bout for respiratory analysis and the final 25 strides of each walking bout for all gait analyses. We normalized all stride-related data from heel-strike (0% gait cycle) to heel-strike (100% gait cycle) of the same leg.

### Metabolic cost

We measured subjects’ *O*_2_ and *CO*_2_ flow rates during walking using a portable open-circuit indirect calorimetry system (CareFusion Oxycon Mobile, Hoechberg, Germany). We converted these measurements to metabolic power using formulas from Brockway [23]. We recorded a three minute standing trial from each subject at the beginning of the testing session and averaged it to get subjects’ standing metabolic work rate. This calculated standing metabolic work rate was then subtracted from each walking bout to calculate the net metabolic work rate [24]. We analyzed each walking bout by averaging the final three minutes of recorded walking metabolic data, and then normalized these averages by subjects’ body mass. During all testing, subjects remained in the aerobic range of exertion as all respiratory exchange ratios were less than one [25].

### Electromyography

We measured muscle activity from the soleus, tibialis anterior, medial gastrocnemius, biceps femoris long head, vastus lateralis, rectus femoris, and gluteus maximus using electromyography (EMG). All EMG recordings and analysis, except for the soleus, came solely from the subjects’ right leg. Soleus activity was recorded and analyzed from both the left and right legs since soleus activity was used as a control input for the proportional myoelectric controller. We recorded all muscle activity using bipolar surface electrodes (sample rate: 1000 Hz; Biometrics, Ladysmith, VA) with an inter-electrode distance of 2.0 cm and electrode diameter of 1.0 cm. The EMG amplifier had a bandwidth of 20-460 Hz. We placed all electrodes on subjects’ legs in accordance to the procedures of Winter and Yack [26].

During post processing, we high-pass filtered all raw EMG signals with a 35 Hz cut-off frequency (3rd order Butterworth filter, zero-lag) and then full-wave rectified the filtered signals. To compute the signals’ linear envelopes, we low-pass filtered the rectified signals with a 10 Hz cut-off frequency (3rd order Butterworth filter, zero lag). Each linear envelope was then parsed by stride (heel-strike to heel-strike), normalized to stride cycle, and averaged. We normalized each muscle’s linear envelope amplitude by its corresponding average peak voltage from the unpowered walking bout on a subject-specific basis [26]. In addition to the linear envelopes, we calculated the root mean square (r.m.s.) stride average for the rectified EMG signals. We normalized each muscle’s r.m.s. stride average by its corresponding average from the unpowered walking bout on a subject-specific basis. All EMG normalization was done prior to averaging.

### Kinematics

All subjects wore a 39 reflective marker set during testing (34 on the pelvis and lower limbs, 4 on the torso, and 1 on the head). We tracked all marker positions using a 10-camera motion capture system (sample rate: 100 Hz; Vicon, Oxford, UK). We calculated joint kinematics from the raw marker data using OpenSim 3.2 [27]. In OpenSim we scaled a generic 23 degree of freedom, 54 actuator model to subject specific marker placements. During processing, we ensured that all subject model scaling and inverse kinematic root mean square values were within the range recommend by OpenSim documentation [28].

We calculated the Pearson product moment correlations between different joint kinematic measurements across different walking bouts. We assessed similarities in joint kinematics by the coefficient of determination (*R*^2^) of these correlations [22]. *R*^2^ values approaching 1 indicate strong similarities in joint trajectories as an *R*^2^ value equal to 1 indicates a perfect match in trajectories. *R*^2^ values close to 0 indicate strong differences in trajectories.

We calculated all gait kinematic measures (step length, step width, step period, double support period) using motion capture data from the left and right calcaneus markers. All gait events were sensed using ground reaction force data from the instrumented treadmill. All raw motion data was first low-pass filtered using a 5 Hz cut-off frequency (3rd order Butterworth filter, zero-lag) to remove any motion artifact. Step length and step width were defined as the fore-aft and lateral distances, respectively, between the calcaneus markers at the time of detected heel strike. Step period was defined as the time between heel strikes of opposite feet, and double support period was defined as the time between heel strike of one foot and the toe off of the other.

### Joint mechanics

To perform inverse dynamics, we imported all ground reaction force data into OpenSim 3.2 and used it in conjunction with the calculated joint kinematics. Each subject model’s mass was scaled anthropomorphically with the manual addition of the mass at the shank and foot to account for the exoskeletons. We removed as much of the residual forces and moments of the inverse dynamics as possible by iteratively adjusting the model using OpenSim’s residual reduction algorithm (RRA). All of the final residuals after using the RRA were within OpenSim’s recommended ranges. They are presented in Table 1 [28].

**Table 1:** Average residual values after final run of the RRA in OpenSim

	*F*_*x*_	*F*_*y*_	*F*_*z*_	*M*_*x*_	*M*_*y*_	*M*_*z*_	pErr _*x*_	pErr _*y*_	pErr _*z*_
	(N)	(N)	(N)	(Nm)	(Nm)	(Nm)	(cm)	(cm)	(cm)
Maximum	9.7	9.4	12.5	27.0	43.4	34.7	2.8	1.8	0.6
Root Mean Square	5.3	2.8	7.4	8.6	21.2	9.7	1.9	1.1	0.3

*F*_*x*_, *F*_*y*_, and *F*_*z*_ refer to the residual forces at the pelvis, and *M*_*x*_, *M*_*y*_, and *M*_*z*_ refer to the residual moments at the pelvis. pErr _*x*_, pErr _*y*_, and pErr _*z*_ refer to the translational position error of the markers

We took the numerical derivative of the joint positions to calculate the joint angular velocities. We filtered these velocities with a 25 Hz cut-off frequency (3rd order Butterworth, zero-lag) to remove any amplified noise that may have resulted from the numerical differentiation. We then multiplied these calculated joint angular velocities by the joint torques resulting from the inverse dynamics to calculate joint power. Exoskeleton power was calculated in a similar fashion, using the calculated ankle angular velocity and the measured actuation torque from the load cell. We subtracted the exoskeleton power from the total ankle power at each time instance to calculate the biological ankle power. Average net joint power was computed by taking the time integral of the power time series data and dividing it by corresponding stride periods [29, 30]. We computed average positive and negative power values in the same way, but by separating out the time integrals to periods of positive and negative power, respectively.

### Exoskeleton mechanics

We measured the distance of the exoskeleton joint center to the actuator attachment point as 10.07 cm. We were able to calculate the moment arm of the actuator at each time instance of collection from this distance measure and the calculated ankle kinematics. We filtered all load cell data with a 25 Hz cut-off frequency (3rd order Butterworth filter, zero-lag) and then multiplied it by this calculated moment arm to compute the exoskeleton torques. To calculate exoskeleton power, we multiplied these torques by the ankle angular velocity. We calculated average exoskeleton power values in the same way as the average joint power values. We calculated exoskeleton mechanics from the left exoskeleton for half of the subjects and the right exoskeleton for the other half due to hardware capabilities during testing.

### Statistical analyses

For all statistical comparisons we performed a paired t-test (*α*=0.05) between walking conditions with the timing-based controller and the proportional myoelectric controller. All tested data was confirmed to be of a normal distribution using a Jarque-Bera test. All reported values and measurements from here forward are presented as the mean ± the standard error of the mean (s.e.m.).

## Results

### Metabolic work rate

**Fig. 3:**
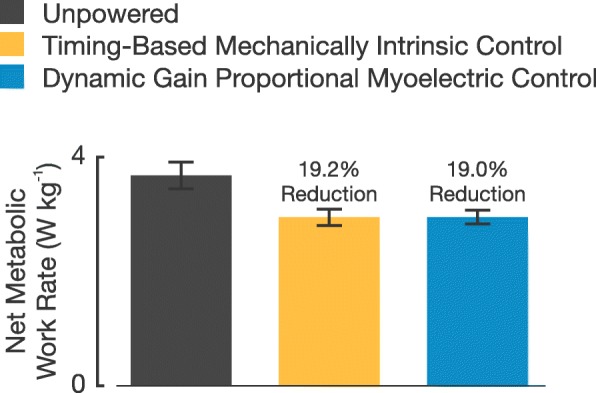
Net Metabolic Work Rate for Each Walking Condition. All net metabolic work rates are normalized to subject mass and represent the absolute changes in energetics for each walking condition. Error bars are ±1 s.e.m. from the mean of each condition. The percentages above each powered condition represents the percent decrease in energetics compared to the unpowered condition

Walking with the exoskeletons powered, regardless of controller used, resulted in large decreases in metabolic work rate compared with the unpowered condition (Fig. 3). Net metabolic work rate of walking in the exoskeletons unpowered was 3.68±0.23 W kg^-1^ (mean ±s.e.m.). Net metabolic work rate of walking with the timing-based mechanically intrinsic controller was 2.95±0.14 W kg^-1^, or a 19.2±2.5*%*, decrease compared to the unpowered condition. Net metabolic work rate of walking with the dynamic gain proportional myoelectric controller was 2.95±0.12 W kg^-1^, or a 19.0±2.5*%*, decrease compared to the unpowered condition. There was no significant difference in metabolic work rates between the timing-based controller and the proportional myoelectric controller (*P*=0.966).

### Electromyography

**Fig. 4:**
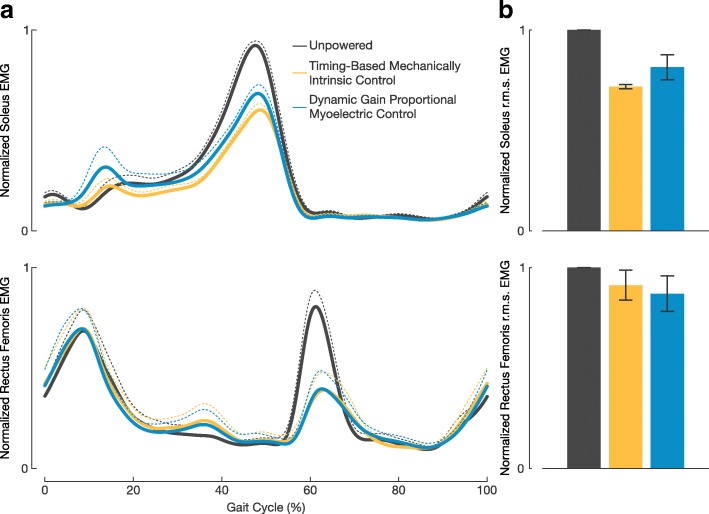
Soleus and Rectus Femoris Electromyography. **a** The mean soleus and rectus femoris EMG linear envelope from each walking condition is represented by the solid lines and +1 s.e.m. is represented by the dashed lines. **b** The mean soleus and rectus femoris r.m.s. of rectified EMG across the three walking conditions. All error bars represent ± 1 s.e.m.

The largest change in muscle activity was observed at subjects’ soleus muscle (Fig. 4). When walking with the timing-based controller, subjects achieved a soleus r.m.s. EMG reduction of 28.2 ± 1.0*%* and a peak linear envelope reduction of 37.5 ± 3.1*%* compared to the unpowered condition. When walking with the proportional myoelectric controller, subjects achieved a soleus r.m.s. EMG reduction of 18.6±6.2*%* and a peak linear envelope reduction of 28.8 ± 4.7*%* compared to the unpowered condition. Subjects soleus r.m.s. EMG was less when using the timing-based controller than when using the proportional myoelectric controller, yet it was not a statistically significant difference (*P*=0.132). There was a distinct qualitative difference in the shape of the two powered walking conditions’ resulting linear envelopes (Fig. 4a). Subjects exhibited a significantly lower peak soleus linear envelope value when using the timing-based controller compared to the proportional myoelectric controller (*P*=0.026). Also, on average subjects showed 11.0±4.9*%* less muscle activity with the timing-based controller compared to the proportional myoelectric controller during the mid and late stance phases of gait (30-50% gait cycle).

Subjects also experienced large reductions in rectus femoris activity during the powered walking conditions compared to the unpowered condition. When walking with the timing-based controller, subjects achieved a rectus femoris r.m.s. EMG reduction of 8.8±7.5*%* and a peak linear envelope reduction of 35.2±20.3*%* compared to the unpowered condition. When walking with the proportional myoelectric controllers, subjects achieved a rectus femoris r.m.s. EMG reduction of 13.0±8.9*%* and a peak linear envelope reduction of 38.6±15.0*%* compared to the unpowered condition. There was little to no difference in the resulting average rectus femoris linear envelopes between the two controllers (Fig. 4a). No statistically significant differences were observed between the two powered conditions resulting r.m.s. EMG values at the tibialis anterior, medial gastrocnemius, biceps femoris long head, vastus lateralis, and gluteus maximus (all *P*>0.05). These additional muscle r.m.s. EMG values and their corresponding statistics are presented in the Additional file 1: Table S1.

### Gait kinematics

There were slight differences between walking conditions’ mean gait kinematics (Table 2). Subjects exhibited slightly larger mean step lengths and step widths when using the timing-based controller than when using the proportional myoelectric controller (*P*=0.004 and *P*=0.030, respectively). There was no statistically significant differences in gait kinematic variability between the two powered conditions (Table 3).

**Table 2:** Resulting mean gait kinematics of each walking bout

Walking condition	Step length	Step width	Step period	Double support period
	(Normalized)	(Normalized)	(ms)	(ms)
Unpowered	0.713 ± 0.010	0.173 ± 0.011	586.3 ± 5.6	161.7 ± 5.0
Timing-Based	0.704 ± 0.007	0.190 ± 0.016	591.6 ± 6.9	167.2 ± 2.8
Proportional Myoelectric	0.692 ± 0.008	0.182 ± 0.014	586.5 ± 6.6	169.0 ± 2.2
*P*-Value	0.004	0.030	0.095	0.256

All values are reported as mean ±s.e.m. across subjects. All distance measurements have been normalized by leg length. *P* <0.05 represents a statistically significant difference between the proportional myoelectric controller and the timing-based controller

**Table 3:** Resulting gait kinematic variability of each walking bout

Walking condition	Step length	Step width	Step period	Double support period
	(Normalized)	(Normalized)	(ms)	(ms)
Unpowered	0.021 ± 0.002	0.018 ± 0.002	14.1 ± 1.2	7.5 ± 0.6
Timing-Based	0.023 ± 0.002	0.021 ± 0.002	17.6 ± 1.5	9.2 ± 0.7
Proportional Myoelectric	0.026 ± 0.004	0.021 ± 0.001	17.1 ± 2.2	11.2 ± 1.1
*P*-Value	0.231	0.970	0.615	0.210

Variability has been defined as the average standard deviation across subjects. All values are reported as mean ±s.e.m. across subjects. All distance measurements have been normalized by leg length. *P* <0.05 represents a statistically significant difference between the proportional myoelectric controller and the timing-based controller

### Joint kinematics

**Fig. 5:**
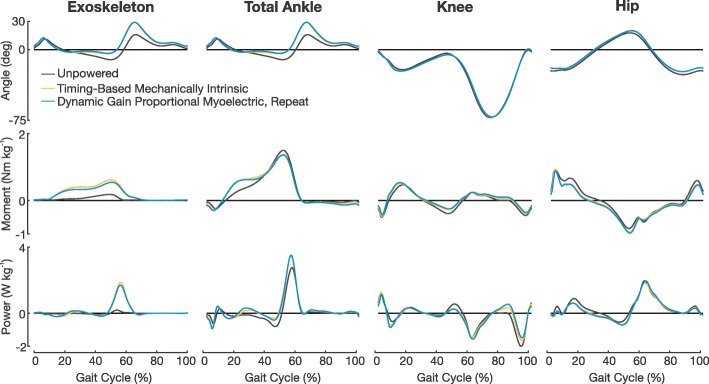
Joint Kinematics, Dynamics, and Power. The mean joint angles, moments, and powers from the unpowered, timing-based mechanically intrinsic control, and second bout with the dynamic gain proportional myoelectric control conditions. Joint dynamics and power have been normalized by subject mass. In the kinematics and dynamics subplots all positive numbers represent extension while all negative numbers represent flexion

During powered conditions, subjects experienced the largest deviations from unpowered walking kinematics at the ankle. Subjects increased plantar flexion by an average ∼14^o^ during the mid-to-late stance phase of gait when using both the timing-based and the proportional myoelectric controllers compared to the unpowered condition (Fig. 5). A linear regression between ankle kinematics of the timing-based controller and of the unpowered condition resulted in an *R*^2^ value of 0.73±0.05. A linear regression between ankle kinematics of the proportional myoelectric controller and of the unpowered condition resulted in an *R*^2^ value of 0.71±0.08. A linear regression between ankle kinematics of the timing-based controller and the proportional myoelectric controller resulted in an *R*^2^ value of 0.98±0.01.

There were little to no changes in joint kinematics at every other joint between conditions. Linear regressions of knee and hip kinematics between powered and unpowered conditions all resulted in *R*^2^ values greater than 0.98. Linear regressions of knee and hip kinematics between the two controllers all resulted in *R*^2^ values greater than 0.99.

### Joint kinetics

Subjects increased their mean total moment at the ankle (biological and exoskeleton) by ∼0.14 Nm kg^-1^ during the early-to-mid stance phase (0-30% gait cycle) when comparing either of the powered conditions to the unpowered condition (an increase of ∼48.9%, Fig. 5). The observed increase in total ankle plantar flexion moment during the early to mid stance phase corresponds with a decrease in hip flexion moment. Subjects decreased their mean hip flexion moment ∼0.12-0.15 Nm kg^-1^ during this phase of the gait cycle when comparing either of the powered conditions to the unpowered conditions (a decrease of ∼25-31%). Subjects also decreased their mean knee extension moment ∼0.08-0.10 Nm kg^-1^ during the mid and late stance phase (30-50% gait cycle) when comparing the powered conditions to the unpowered condition (a decrease of ∼31-42%).

**Fig. 6:**
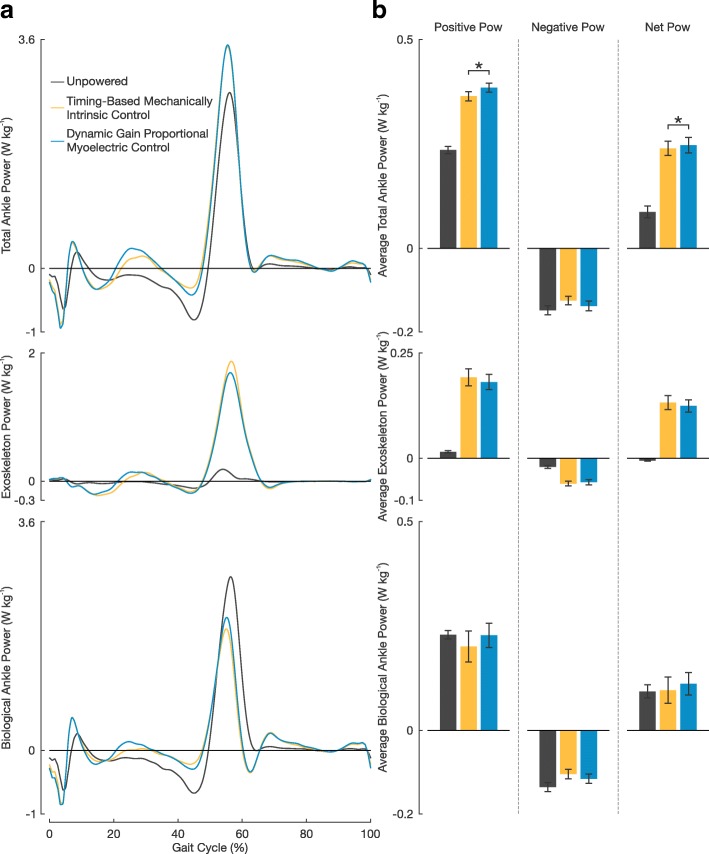
Ankle Power Contributions. **a** Mean total ankle power, exoskeleton power, and biological ankle power across the three walking conditions. The exoskeleton power was calculated from ankle kinematics and force outputs recorded using the exoskeletons’ load cells. The biological power was calculated by subtracting the exoskeleton power from the total ankle power. **b** Average power plots of positive, negative, and net power for total ankle power, exoskeleton power, and biological ankle power. All error bars represent ±1 s.e.m. An asterisk above the plots represents a significant difference between the two powered walking conditions (*P*<0.05)

Subjects showed large increases in positive and net average total ankle power when the exoskeletons were powered compared to unpowered (Fig. 6). When using the timing-based controller, subjects had an average positive total ankle power 0.13±0.01 W kg^-1^ and an average net total ankle power 0.15±0.01 W kg^-1^ larger than that when walking in the devices unpowered (an increase of 55.2±4.0*%* and 213.0±38.5*%*, respectively). When using the proportional myoelectric controller, subjects had an average positive total ankle power 0.15±0.01 W kg^-1^ and an average net total ankle power 0.16±0.01 W kg^-1^ larger than that when walking in the devices unpowered (an increase of 64.0±3.8*%* and 222.9±42.3*%*, respectively). Subjects showed significantly larger average positive and negative total ankle power when using the proportional myoelectric controller compared to when using the timing-based controller (*P*=0.005 and *P*=0.001, respectively). There was no statistically significant difference in average positive, negative, or net exoskeleton power output between the two controllers (*P*=0.124, *P*=0.313, and *P*=0.138, respectively). There was also no statistically significant difference in average positive, negative, or net biological ankle power output between the two controllers (*P*=0.056, *P*=0.102, and *P*=0.057, respectively); however, the difference in average positive and net biological ankle power were near significant. Subjects on average achieved ∼0.18 W kg^-1^ greater exoskeleton peak power when using the timing-based controller than when using the proportional myoelectric controller (an increase of ∼11.1%). There was a statistically significant difference between these peak power values (*P*=0.048). This increase in peak exoskeleton power corresponded with an average decrease in peak biological ankle power of ∼0.11 W kg^-1^ when using the timing-based controller compared to the proportional myoelectric controller (a decrease of ∼8.3%). Due to large variability in subject data, this observation was not of a statistically significant difference (*P*=0.439).

**Fig. 7:**
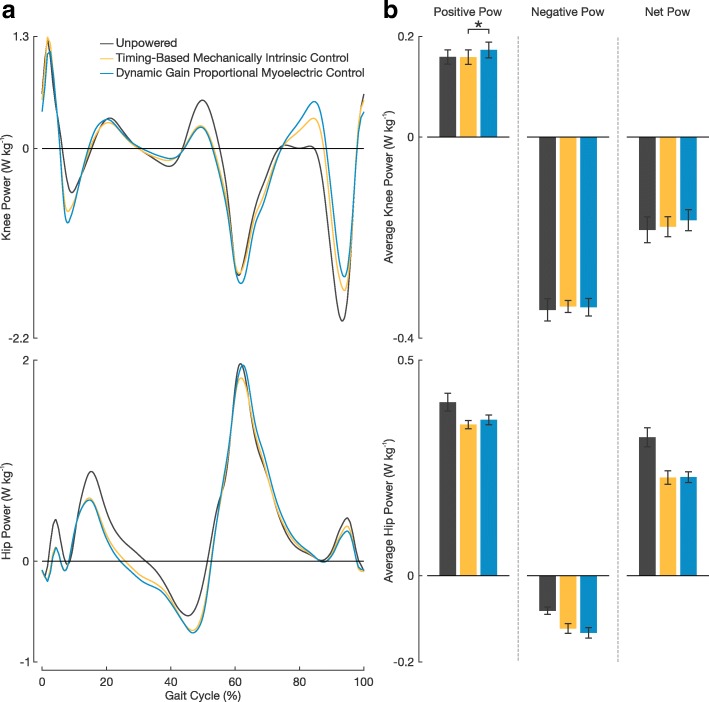
Knee and Hip Power Contributions. **a** Mean knee power and mean hip power across the three walking conditions. **b** Average power plots of positive, negative, and net power at the knee and hip. All error bars represent ± 1 s.e.m. An asterisk above the plots represents a significant difference between the two powered walking conditions (*P*<0.05)

Subjects put forth significantly greater positive average knee power when using the proportional myoelectric controller than when using the timing-based controller (*P*=0.003, Fig. 7). There were no statistically significant differences in negative or net positive power at the knee between the two controllers (*P*=0.851 and *P*=0.063, respectively). Subjects showed large differences in average negative and net power at the hip between powered and unpowered conditions. When using the timing-based controller, subjects had an average negative hip power 0.04 ± 0.01 W kg^-1^ and an average net hip power 0.09±0.02 W kg^-1^ larger than that when walking in the devices unpowered (an increase of 52.3±5.0*%* and 28.1±4.1*%*, respectively). When using the proportional myoelectric controller, subjects had an average negative hip power 0.05±0.01 W kg^-1^ and an average net hip power 0.09±0.01 W kg^-1^ larger than that when walking in the devices unpowered (an increase of 66.4±9.8*%* and 28.0±2.5*%*, respectively). There was no statistically significant difference in average positive, negative, or net hip power between controllers (*P*=0.232, *P*=0.057, and *P*=0.934, respectively).

## Discussion

Our primary hypothesis was that metabolic work rate would differ between walking with the timing-based mechanically intrinsic controller and the dynamic gain proportional myoelectric controller. However, we found quite the opposite. Results show that there was no statistically significant difference in metabolic work rate between the two control strategies in this experiment (Fig. 3). The reasoning for why we expected a different metabolic work rate between controllers was that we expected subjects to use each controller in a unique way due to the fact that one control strategy was dependent on muscle recruitment and the other was not. Although we did not observe a difference in metabolic work rate, we did observe differences in other biomechanical measures.

Soleus muscle recruitment differed between walking with the proportional myoelectric controller and with the timing-based controller. Although there was not a statistically significant difference in soleus r.m.s. EMG values between the two walking conditions, there was a strong trend in subjects using less soleus muscle recruitment when using the timing-based controller than when using the proportional myoelectric controller. This was made evident by the absolute values of the r.m.s. EMG calculations and the fact that subjects’ resulting soleus linear envelopes when using the timing-based controller were 11% less than that of the proportional myoelectric controller during the mid and late stance phases of gait (Fig. 4a). Additionally, the peak value of subjects’ soleus linear envelopes when using the timing-based controller was significantly less than that when using the proportional myoelectric controller. Similar trends were observed in medial gastrocnemius r.m.s. EMG calculations as well (Additional file 1: Table S1). We believe the reason we did not observe differences in metabolic work rate in this study despite this difference in muscle recruitment was that these devices targeted a relatively small muscle group. If repeated with an exoskeleton that targeted larger muscle groups, such as with a hip exoskeleton [31–33], it might be expected to see differences in metabolic work rate; however, further research is needed to make this conclusion.

The observed differences in recruitment of plantar flexor muscles had a direct effect on resulting ankle mechanics. We observed that subjects walked with significantly larger average positive and negative total ankle power when using the proportional myoelectric controller than when using the timing-based controller (Fig. 6). This increase in power magnitude at the ankle is due to the very slight differences in total ankle moment of the two controller conditions (Fig. 5). In looking at the breakdown of total ankle power contributions we see that subjects trended toward significantly larger peak exoskeleton power output when using the timing-based controller than the proportional myoelectric controller (an increase of 11.1%). This corresponded with a trend in decreased peak biological ankle power output (a decrease of 8.3%). Additionally, subjects used less average positive and net biological ankle power when walking with the timing-based controller than with the proportional myoelectric controller. These differences in average positive and net biological ankle power were near statistical significance (*P*=0.056 and *P*=0.057, respectively). These ankle power results suggest that when using the timing-based controller subjects were contributing less to locomotion at the biological ankle and are more so ‘along for the ride’ [34]. This makes sense given that active engagement and involvement at the ankle is not necessarily required when using the timing-based controller. So long as heel strike occurs, subjects will receive actuation. When using the proportional myoelectric controller, active involvement on a muscular level is necessary to directly control the actuation of the device. Additionally, these changes in ankle dynamics seemed to have had an effect on subjects average positive knee power as subjects walked with significantly more positive knee power when using with the proportional myoelectric controller than when using with the timing-based controller.

We attribute these differences in muscle recruitment, and thus the resulting observed differences in ankle power, to the theory of slacking. The idea behind slacking is that the human motor system is always trying to minimize its levels of muscle activation during repetitive tasks where movement error is small, such as walking [34, 35]. One hypothesis for interpreting the differences in soleus muscle recruitment between the two controllers is that when using the proportional myoelectric controller, users can only slack so far before signal quality affects walking stability. This is an inherent technical limitation of using measures of muscle activity to drive a controller. As mentioned prior, neural signals, such as muscle activity, often have large noise content. Therefore as subjects decrease their muscle activity, the signal-to-noise ratio of the measurement decreases making it difficult to separate the signal from the noise. In this specific experiment, the dynamically adjusting gain of the proportional myoelectric controller increases to compensate for decreases in muscle activity. This larger gain will amplify any noise that makes it through the filtering process along with the intended control signal. This amplified noise could then make control of the device difficult and potentially cause for instability with walking. Given this argument, we believe there is a maximum level of slacking that can be obtained with the proportional myoelectric controller presented here. When using the timing-based controller, the actuation is consistently the same for each step regardless of muscle activity. Because of this, users can potentially slack their muscle activity further than with the proportional myoelectric controller without any change to actuation.

Another hypothesis for interpreting the differences in soleus muscle recruitment between the two controllers is that when using the proportional myoelectric controller, users can only slack so far due to the means in which the controller is triggered. No matter what, subjects must use some amount of muscle recruitment to actuate the proportional myoelectric controller. Due to the fact that this controller inherently guarantees synchronous actuation with the user, the user is always moving with the device during actuation. When using the timing-based controller, subjects do not necessarily need to move with the device. They could potentially lean into the actuation and let it propel them forward in a way that is not possible with the proportional myoelectric controller. This could potentially explain the observed increases in peak exoskeleton power and decreases in biological ankle power. Once figuring out this strategy, subjects can exploit it and become disengaged at a muscle level during walking. Thus, subjects are able to slack further when using the timing-based controller.

Equally interesting to the resulting differences in these two control strategies are how in which the two were similar. The results from this study show that regardless of the control strategy being used, the actuation from the exoskeletons resulted in large reductions the user’s metabolic work rate compared to walking unpowered in the devices. The absolute value of these reductions is comparable to previous work in the field [1, 3, 4, 19]. Although it is not a novel finding to show that actuation of an exoskeleton can reduce the metabolic work rate compared to unpowered walking, it is a good proof that both control strategies were able to work in parallel with user to offload some of the energetic requirements of walking. Additionally, gait kinematics between the two controllers were relatively unchanged (Tables 2 and 3). We found that all lower extremity joint kinematics were largely unchanged from one controller to the other, as evident that all regressions between the two controllers resulted in *R*^2^ values greater than 0.98 (Fig. 5). We also observed that regardless of the controller being used, subjects adapted to large increases in total ankle powered compared to the unpowered walking condition. This large increase in total ankle power corresponded with large reductions in power at the hip. These reductions in hip power were congruent with reductions in EMG activity at the rectus femoris (Fig. 4b). This trade off in joint power and muscle activity between the ankle and hip is consistent with previous work by our research group and that observed by others with different devices and controllers [4, 19, 36]. We find all of these resulting similarities between the two controllers an interesting finding as they show that if a timing-based controller is designed properly and used under the appropriate walking constraints, the resulting biomechanical and energetic adaptations mimic that of the proportional myoelectric controller. With the correct design, a researcher could potentially use either type of control scheme with an ankle exoskeleton and achieve largely the same results for steady-state walking; the major differences being in the resulting ankle muscle recruitment and ankle mechanics.

It is difficult to pinpoint the reasons behind the biomechanical differences observed between the two controllers, but the fact that they exist lend insight to when one controller may be better suited than another. For example, if a device is targeted toward therapeutic rehabilitation of neurological injuries [37], a controller driven by neural signals may be more beneficial than one driven by mechanically intrinsic signals due to users being more engaged at a muscle level when using a controller driven by neural signals. This suggestion is drawn from the success of gait training with human therapists being more successful than that with robotic devices in patients with chronic stroke [38]. This difference is attributed to patients’ active involvement when working with a therapist over a robotic device. As this study has only considered testing with healthy subjects, further research would need to be conducted with a clinical population to draw definitive conclusions on this. Additionally, the results from this study suggest that if a metabolic reduction is of interest, it appears that either type of control strategy could be employed.

We acknowledge that this study is a comparison of a single controller driven by neural signals and a single controller driven by mechanically intrinsic signals. There are an infinite number of possible controllers for each that could be compared; however, we believe this is a strong starting point for future work in better understanding controller design for specific applications. Seeing as each control strategy lends itself to different pros and cons, a hybrid of the two approaches may be advantageous, an area of research many have already begun exploring [39–42]. We also acknowledge that this experiment was performed with a young, able-bodied population walking in a straight line at a constant velocity with a hardware platform that was limited in torque output. Further research would need to be performed to show how these principles hold with different populations, devices, and walking scenarios.

## Conclusion

This study aimed to compare the differences between walking in bilateral ankle exoskeletons using a dynamic gain proportional myoelectric controller and using a timing-based mechanically intrinsic controller. We hypothesized that these two controllers would result in different measures of metabolic work rate due to expected differences in biomechanical measures. We observed no differences in metabolic work rate, small changes in joint kinetics at the knee and hip, and virtually no difference on all leg joint kinematics between the two controllers. As such, we can conclude that if designed properly and under the appropriate walking constraints, the timing-based controller can adequately mimic the proportional myoelectric controller. The major differences between these two controllers that we did observe were at the ankle. Subjects showed increased soleus muscle activity when using the proportional myoelectric controller than when using the timing-based controller. This corresponded with significantly larger positive and net total ankle power when using the proportional myoelectric controller than when using the timing-based controller. These findings suggest that a controller driven by neural signals may be better suited for therapeutic rehabilitation applications while either controller is well suited for human augmentation purposes.

## Additional file

Additional file 1 **Table S1**. Root mean square electromyography data for all recorded muscles. (PDF 64.1 kb)
