# Supplementary material for: Biomechanics and energetics of walking in powered ankle exoskeletons using myoelectric control versus mechanically intrinsic control
Source: J Neuroeng Rehabil. 2018 May 25;15:42. doi: 10.1186/s12984-018-0379-6 (PMC5970476; doi:10.1186/s12984-018-0379-6)
Supplement: Supplementary file 1 — Table S1. Root mean square electromyography data for all recorded muscles. (PDF 64.1 kb) [file 12984_2018_379_MOESM1_ESM.pdf]

Table S1 Root mean square electromyography for muscles of interest

	Unpowered	Timing-Based Mechanically Intrinsic	Dynamic Gain Proportional Myoelectric	$P-$Value
Soleus r.m.s.				
	1.00 ± 0.00	0.72 ± 0.01	0.81 ± 0.06	0.132
Tibialis Anterior r.m.s.				
	1.00 ± 0.00	0.94 ± 0.04	0.89 ± 0.05	0.087
Medial Gastrocnemius r.m.s.				
	1.00 ± 0.00	0.84 ± 0.06	0.89 ± 0.07	0.423
Biceps Femoris Long Head r.m.s.				
	1.00 ± 0.00	0.87 ± 0.07	0.78 ± 0.08	0.156
Vastus Lateralis r.m.s.				
	1.00 ± 0.00	1.05 ± 0.03	0.98 ± 0.04	0.249
Rectus Femoris r.m.s.				
	1.00 ± 0.00	0.91 ± 0.07	0.87 ± 0.09	0.394
Gluteus Maximus r.m.s.				
	1.00 ± 0.00	0.86 ± 0.04	0.85 ± 0.04	0.550

Values (means ± s.e.m.) are root mean square (r.m.s.) average from the whole walking stride normalized to the unpowered condition. $P<0.05$ represents a statistically significant difference between the two powered walking conditions.
